# Gait analysis with smart insoles can identify patients at risk of tibial shaft fracture nonunion as early as six weeks after surgery: longitudinal and cross-sectional study

**DOI:** 10.3389/fbioe.2025.1536738

**Published:** 2025-06-25

**Authors:** Elke Warmerdam, Marcel Orth, Max Müller, Tim Pohlemann, Bergita Ganse

**Affiliations:** ^1^ Werner Siemens-Endowed Chair for Innovative Implant Development (Fracture Healing), Departments and Institutes of Surgery, Saarland University, Homburg, Germany; ^2^ Department of Trauma, Hand and Reconstructive Surgery, Departments and Institutes of Surgery, Saarland University, Homburg, Germany

**Keywords:** bone regeneration, diaphyseal fracture, fracture monitoring, orthopaedic trauma, patient-reported outcome measures, plantar pressure, postoperative monitoring, wearables

## Abstract

**Background:**

Nonunion, a common and detrimental complication of tibial shaft fractures, is usually diagnosed via X-ray-based imaging. Unfortunately, it often takes months of observation until the indication for revision surgery or other interventions is given, which is why additional methods are desirable to enhance the ability to predict and prevent nonunion earlier.

**Objective:**

As gait patterns and subjective outcomes obtained by questionnaires improved during regular fracture healing, the aim of this study was to determine whether gait analyses with instrumented insoles and patient-reported outcome measurement information system (PROMIS) questionnaires could be used to detect patients at risk of developing nonunion during the healing phase after tibial shaft fracture.

**Methods:**

Data were collected from a longitudinal and a cross-sectional tibial fracture cohort via gait analysis with instrumented insoles (OpenGO, Moticon GmbH, Munich, Germany) and in addition via PROMIS questionnaires. The gait parameters included pressure, temporal, angular velocity and acceleration-related parameters. The PROMIS covered the global health, physical function and pain questionnaires. Comparisons between patients with and without union were performed at 1 week, 6 weeks, 3 months and 6 months after surgery.

**Results:**

Six-month postoperative radiographs revealed nonunion in seven of 18 longitudinally assessed patients and in four patients who underwent a single assessment after nonunion diagnosis. Overall gait patterns, primarily reflected by temporal and pressure distribution parameters, differed significantly between patients with and without union from 6 weeks onward. These differences between union and nonunion patients were detected via gait patterns significantly earlier than by radiographs or PROMIS questionnaires. In detail, 6 weeks after surgery, 16 out of the 33 gait parameters were significantly different between the longitudinal union and longitudinal nonunion groups. Three months after surgery, the center of pressure width (p = 0.022), stride time (p = 0.035), stride frequency (p = 0.008), maximal angular velocity (p = 0.014), and asymmetry of the maximal angular velocity (p = 0.035) differed significantly between the longitudinal union and longitudinal nonunion groups.

**Conclusion:**

Gait analysis with instrumented insoles can be used to detect patients at high risk of developing nonunion of a tibial shaft fracture already 6 weeks postoperative.

## 1 Introduction

Nonunion is a complication of tibial shaft fractures that occurs in approximately 7.6% of patients after treatment (Zura et al., 2016). It is associated with high socioeconomic costs (Hak et al., 2014; Rupp et al., 2018). Several types of nonunion exist and have differing underlying causes, including hypertrophic and atrophic nonunion caused by either biomechanical (e.g., instability and excess forces) or physiological (e.g., lack of blood supply) factors, respectively (Rupp et al., 2018). Nonunion is currently diagnosed on the basis of radiographs and other X-ray-based imaging technologies, such as computed tomography, which are acquired infrequently and are associated with radiation exposure resulting in cancer risk. Moreover, with radiographic assessments, it can take months before it is certain that the fracture is not healing because callus mineralization of the fracture gap is delayed compared with callus stiffening (Blokhuis et al., 2001). Therefore, nonunion is usually not diagnosed until six to 9 months after the injury (Qvist et al., 2021; Özkan et al., 2019). During this period and the several months or even longer after diagnosis and revision surgery until union is achieved, patients have reduced physical and mental health as well as increased pain, leading to a lower quality of life (Brinker et al., 2013).

The treatment of nonunion can be a long and difficult process. There are invasive and noninvasive options available. The invasive options include, among others, the dynamization of a tibial nail. During nail dynamization, one or more screws that hold the nail in place are removed, while other screws that are positioned in gliding holes remain. This exerts compression on the fracture under load which results in more forces and movement in the fracture gap, or exchanging or augmenting the implant in different ways (Bowers and Anderson, 2024). In addition, fracture revision with a bone graft is a popular intervention (Gómez-Barrena and Ehrnthaller, 2024). These invasive surgical modifications can improve the biomechanical conditions for fracture healing and stimulate vascularization. Noninvasive options known to improve bone healing include low-intensity pulsed ultrasound (Harrison and Alt, 2021; Leighton et al., 2021), extracorporeal shockwave therapy (Birnbaum et al., 2002) and pulsed electromagnetic fields (Assiotis et al., 2012). If nonunion could be predicted earlier, these invasive and noninvasive therapies could be applied sooner, leading to earlier healing success. This is likely to decrease impairments in mobility, improve quality of life and reduce health care costs (Hak et al., 2014; Bell et al., 2016; Antonova et al., 2013).

Gait analysis is a method that could help to detect nonunion earlier. The gait pattern is well known to improve over time in patients with a regularly healing lower leg fracture (Warmerdam et al., 2023); specifically, in patients with tibial shaft fractures, which carry the highest risk of nonunion among lower leg fractures, factors known to improve after surgery include spatiotemporal gait (asymmetry) parameters, plantar pressure parameters, kinematics and kinetics (Kröger et al., 2022; Lajevardi-Khosh et al., 2019; Larsen et al., 2017). In a study where one out of multiple patients developed a nonunion or in patients with unsatisfactory long-term results, deviations in pressure-related gait parameters were visible compared to patients experiencing union or satisfactory long-term results (Lajevardi-Khosh et al., 2019; Becker et al., 1995). This indicates that gait parameters could have the potential to help identify patients experiencing nonunion; however, evidence on how gait patterns change in patients who will develop nonunion is lacking.

Additional outcome parameters increasingly used in patients with fractures that reflect the subjective patient perspective are calculated from the questionnaire-based patient-reported outcome measurement information system (PROMIS) (Houwen et al., 2022). Among these, physical function and pain interference are the most frequently used PROMIS scores (Houwen et al., 2022), and the global health questionnaire was also found to be suitable for orthopaedic trauma (O’Hara et al., 2020). Physical function scores were associated with range of motion after a distal humeral fracture (Bhashyam et al., 2020). In patients with tibial fractures, pain interference scores were poorer in patients with lower physical function (Wheelwright et al., 2022). In addition, patients with an orthopaedic infection had worse physical function and pain interference scores (Lee et al., 2023). It is currently unknown how the PROMIS scores change throughout tibial fracture healing; however, they have the potential to aid in the diagnosis of nonunion.

The aim of this study was to analyse whether gait analysis and PROMIS can be used to detect patients who are at risk of developing nonunion from tibial shaft fractures. This retrospective study analysed patients with longitudinal tibial shaft fractures, several of whom developed nonunion. Additionally, patients diagnosed with nonunion were measured cross-sectionally. Differences in gait parameters between longitudinal patients with or without union were already present at 6 weeks after surgical treatment. The earlier patients at risk of developing nonunion are detected, the earlier additional treatment can be started to improve long-term outcomes.

## 2 Methods

### 2.1 Study design

This study comprises a prospective longitudinal and a cross-sectional observational cohort with gait analyses and questionnaires administered to patients with tibial shaft fractures. In the longitudinal group, gait patterns and patient-reported outcome measures were recorded during each routine clinical visit to the University Hospital. Six months after surgery, the patients were diagnosed with either union or nonunion. Differences in gait and patient-reported outcome measures between the union and nonunion groups were analysed at multiple timepoints throughout the healing process.

To confirm differences in gait patterns between patients with union or nonunion, patients who were referred to the nonunion outpatient clinic with nonunion of the tibial shaft were recruited to increase the sample size. The patients’ gait patterns were recorded once and compared to the gait data of patients showing union in the prospective longitudinal observational study at 6 months after surgery.

### 2.2 Ethics approval

The study protocol was approved by the Institutional Review Board of Saarland Medical Board (Ärztekammer des Saarlandes, Germany, application number 30/21). This study was conducted in accordance with the Declaration of Helsinki. All patients provided written informed consent. The study was registered with the German Clinical Trials Registry (DRKS-ID: DRKS00025108).

### 2.3 Participants

Two patient groups, the longitudinal group and the cross-sectional nonunion (CSN) group, were recruited at Saarland University Hospital between February 2022 and January 2024. The longitudinal group comprised patients who underwent surgery to treat tibial shaft fractures and who were prospectively recruited. During the surgeries, these patients either received a plate with screws or a tibial nail according to the recommendations of the AO Foundation (AO foundation 2025). If patients required additional invasive treatment other than tibia nail dynamization, e.g., because of an infection or implant failure, only the data before the revision surgery were taken into account. The exclusion criteria were age younger than 18 years, pregnancy, inability to give consent, use of walking aids before the fracture, other injuries, or disorders that affect walking.

The CSN group comprised patients with nonunion who were referred to the outpatient clinic of Saarland University Hospital and were assessed once at the time of arrival to the outpatient clinic. The inclusion criteria were nonunion of a tibial shaft fracture and surgical treatment less than 18 months prior. The exclusion criteria for the longitudinal patient group were also applied to the CSN group.

### 2.4 Acquisition of reference radiograph images

Radiographs were taken as part of routine clinical care. For the longitudinal group, radiographs obtained approximately 6 months after surgery were judged by physicians as either showing union or showing nonunion. A nonunion diagnosis was made when there was a lack of callus bridging visible on the radiographs. For the CSN group, radiographs obtained upon arrival at the nonunion outpatient clinic were judged by a physician (authors: MO or MM) as either showing union or showing nonunion. Only data from patients whose radiographs showed nonunion were included in the CSN group.

### 2.5 Acquisition of gait data

**FIGURE 1:**
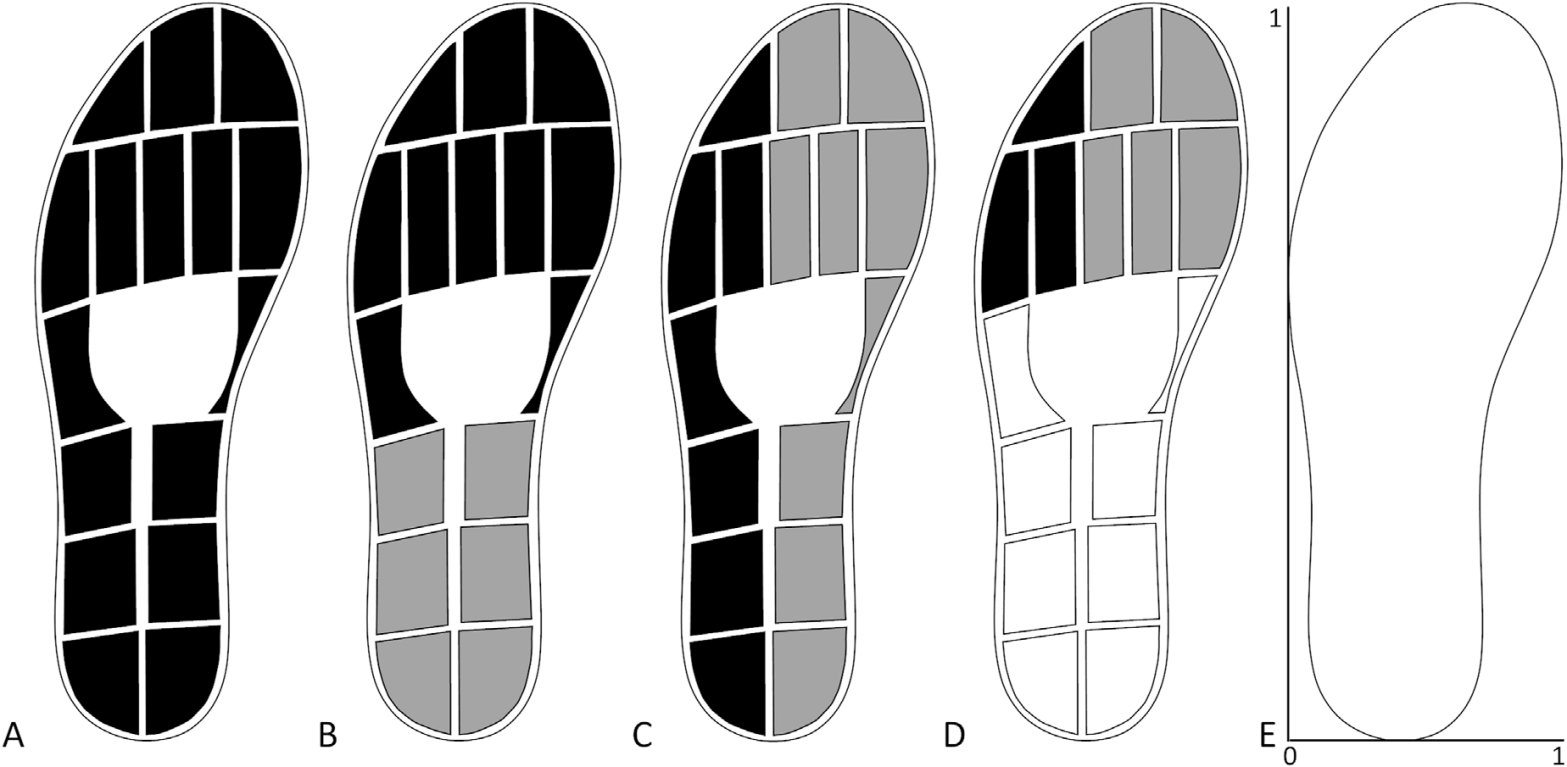
Sensor layout of insoles and combination of sensors used to extract the gait parameters. **(A)** Total force. **(B)** Forefoot (black) and hindfoot (grey) pressure. **(C)** Lateral (black) and medial (grey) pressure. **(D)** Lateral (black) and medial (grey) forefoot pressure. **(E)** Scaling for the centre of pressure measures.

All patients in both groups underwent a gait analysis with instrumented insoles containing 16 pressure sensors (Figure 1A), a triaxial accelerometer and a triaxial gyroscope (OpenGO insoles, Moticon GmbH, Munich, Germany). Insoles were assigned to each individual according to their shoe size. Before each measurement, the insoles were calibrated according to the insole software protocol. The data were sampled at 100 Hz. For each measurement, patients walked in a straight line for 10 m at their preferred gait speed.

For the longitudinal group, gait analyses were performed during the patients’ inpatient stay after surgery and during each follow-up visit. The inpatient measurement of gait parameters was carried out as soon as the patient could walk with crutches and was allowed to put weight on the foot on the injured side. Follow-up visits occurred when routine clinical examinations were scheduled by the treating physician. These examinations generally took place approximately 6 weeks, 3 months and 6 months postoperatively, although deviations from this scheme were frequent. For the CSN group, gait parameters were measured only once at the time of arrival at the outpatient clinic.

### 2.6 Acquisition of the patient-reported outcome measures

To analyse whether patient-reported outcome measures could be used to discriminate between patients with or without union, patients in the longitudinal group were asked to complete three PROMIS questionnaires during each outpatient visit. These were the global health (Scale v1.2) questionnaire, which takes both mental and physical health into account. The physical function short form (Item Bank v2.0) questionnaire, which assesses the self-reported ability to perform various physical activities. The pain interference short form 8a (Item Bank v1.0) questionnaire which assesses the self-reported consequences of pain (PROMIS, 2024).

### 2.7 Analysis of gait data

The data obtained by the instrumented insoles were preprocessed by applying a fourth-order Butterworth filter with a 6 Hz cut-off frequency to remove noise from the data. To enable comparison of the data between patients, the vertical force data and the single pressure sensor data were normalized on the basis of the patient’s body weight and were expressed as percentage body weight and percentage body weight per cm^2^, respectively. Then, the vertical force data were used to detect the initial and final contact of a stride. The onset of the first period of 300 ms or longer, during which the nonnormalized force was less than 30 N, was defined as the final contact. Similarly, the first instant at which the force exceeded 30 N after the final contact was detected was defined as the initial contact. Five strides for each side, the injured and uninjured sides, were extracted from the middle part of the 10 m straight walk measurement. The first and last strides were discarded to minimize the effects of gait initiation and termination on the results.

The total force, single pressure sensor data and centre of pressure (COP) data were obtained with the software provided with the insoles. The maximal force during each stand phase was extracted from the total force (Figure 1A). The forefoot and hindfoot pressures were calculated on the basis of the ten sensors at the front and the six sensors on the back halves of the foot, respectively (Figure 1B). The lateral and medial pressures were calculated on the basis of the seven sensors on the lateral side and the nine sensors on the medial side, respectively (Figure 1C). The lateral and medial forefoot pressures were calculated on the basis of the three and five sensors on the lateral and medial forefoot, respectively (Figure 1D). To correct for the different number of sensors used to calculate the pressure underneath different parts of the feet, the pressure data were divided by the number of sensors after they were summed. This signal was used to extract the maximum pressure per stand phase. The COP length and width were calculated by subtracting the minimum position from the maximum position in the anteroposterior direction and mediolateral direction, respectively. The COP position was the average position of the COP calculated in both the anteroposterior direction and mediolateral direction (Figure 1E). The temporal gait parameters extracted on the basis of the initial and final contacts were the percentage stance time, percentage swing time, stride duration, and stride frequency. From the accelerometer data, the maximum vertical acceleration of each stride, which occurs shortly after the initial contact, was extracted. The maximum angular velocity around the mediolateral axis, which occurs during the push-off phase, was also extracted for each stride. The averages across strides for the injured side parameters were used for further analysis, as well as the differences between the averages of the injured and uninjured sides as asymmetry measures. The asymmetry of each parameter for each patient was calculated as a percentage with the following equation:

$$Asymmetry=\frac{\left(uninjured\;side-injured\;side\right)}{\left(0.5*\left(uninjured\;side+injured\;side\right)\right)}*100$$

### 2.8 Statistical analysis

Statistical analysis was performed with JASP (version 0.17.3, Amsterdam, Netherlands). The demographic and clinical data extracted from the patient records of the longitudinal patient group with union (LU group), longitudinal patient group with nonunion (LN group) and CSN group were compared using one-way ANOVA. To compare the gait parameters collected 1 week, 6 weeks, and 3 months after surgery from the LU and LN groups, Mann‒Whitney U tests were performed because of the small sample size. The gait parameters of the LU, LN and CSN groups at 6 months after surgery were compared using one-way ANOVA if data were normally distributed according to a Shapiro-Wilk test. If the homogeneity of variance was violated, a Brown-Forsythe correction was used. If the assumption of normal distribution was violated the comparison was done with Kruskal–Wallis tests. Post hoc tests were performed with Tukey corrections in case of normal distribution otherwise Dunn’s method was used. The PROMIS results at 6 weeks, 3 months, and 6 months were compared between the LU and LN groups using the Mann‒Whitney U test. A p value <0.05 was considered to indicate significance in all tests.

## 3 Results

### 3.1 Patient demographics

**FIGURE 2:**
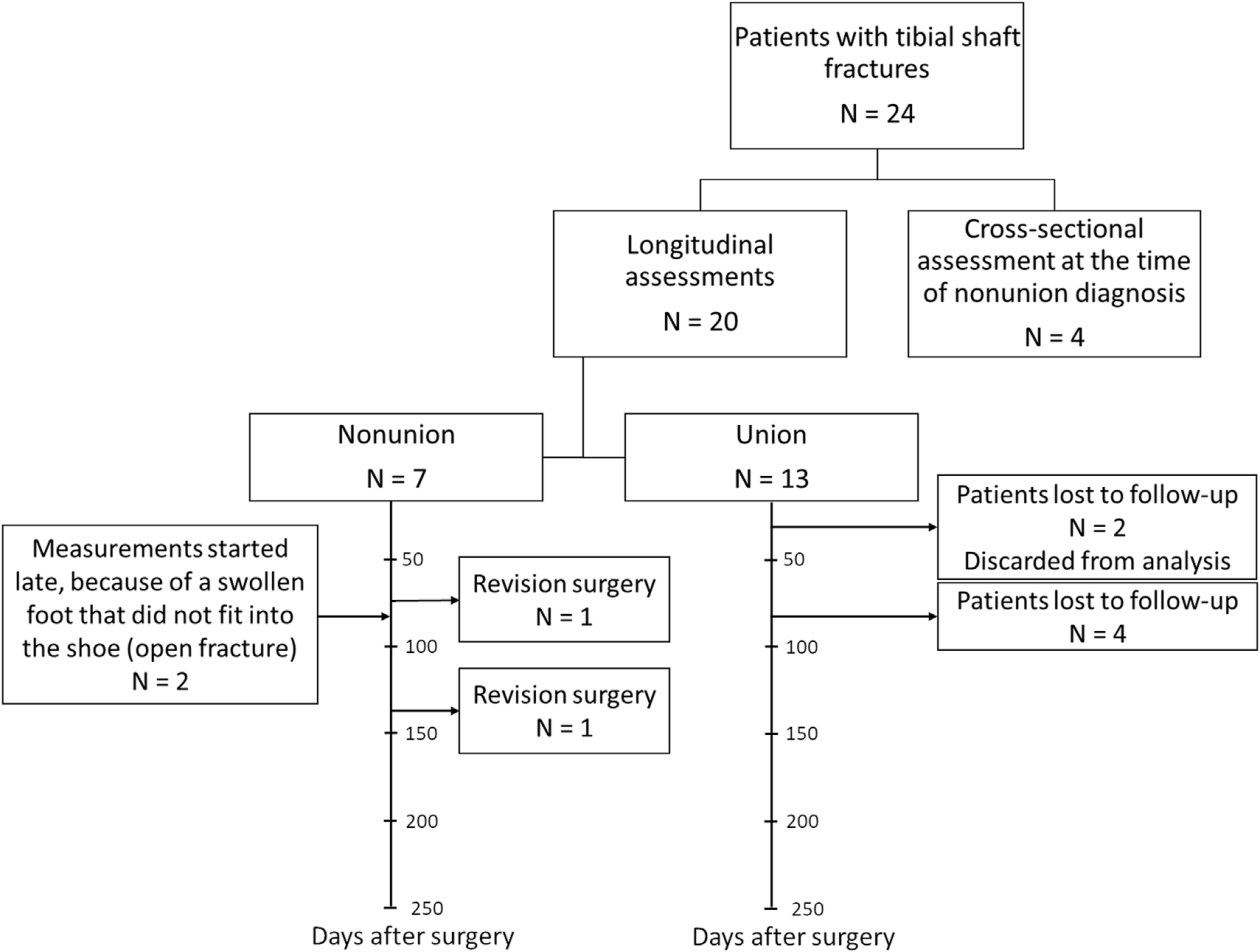
Patient recruitment flowchart per patient group with timeline for the longitudinal union and nonunion groups.

Twenty-four patients were recruited in total (Figure 2). Four patients were included in the CSN group (with the mean time of diagnosis being 10 months after surgery). Twenty patients were enrolled in the longitudinal group. Seven of these 20 patients developed nonunion. Two of the LN patients, one with an infection and one with broken screws, underwent revision surgery within the follow-up period. Data obtained after revision surgery were not included in the analysis. Two other LN patients were not assessed shortly after surgery because the foot was too swollen after an open fracture to fit into a shoe with the instrumented insole; however, they were measured during follow-up assessments. In the LU group, two patients were lost to follow-up after the first measurement; because there were no longitudinal data for these patients, they were excluded from the analysis. Another four patients in this group either did not attend the 3-month follow-up appointment or switched to a local healthcare facility. The demographics of patients in the LU, LN and CSN groups were similar; only the type of implant significantly differed between the groups (Table 1). All patients who achieved union had received a nail.

**TABLE 1:** Patient demographic and clinical information.

	Longitudinal group		Cross-sectional group	
	Union (LU group)	Nonunion (LN group)	Nonunion (CSN group)	P value (ANOVA or χ^2^ test)
N (M:F)	11 (6:5)	7 (6:1)	4 (2:2)	
Age (years)	44 ± 15	51 ± 21	53 ± 24	0.629
Height (cm)	179 ± 8	183 ± 8	177 ± 12	0.530
Weight (kg)	85 ± 15	87 ± 9	73 ± 15	0.238
Smoking N (%)	0 (0)	2 (29)	2 (50)	0.059
Diabetes N (%)	1 (9)	2 (29)	0 (0)	0.341
Underwent surgical fixation with a plate/nail	0/11	3/4	2/2	0.038

### 3.2 Longitudinal union vs. longitudinal nonunion

**FIGURE 3:**
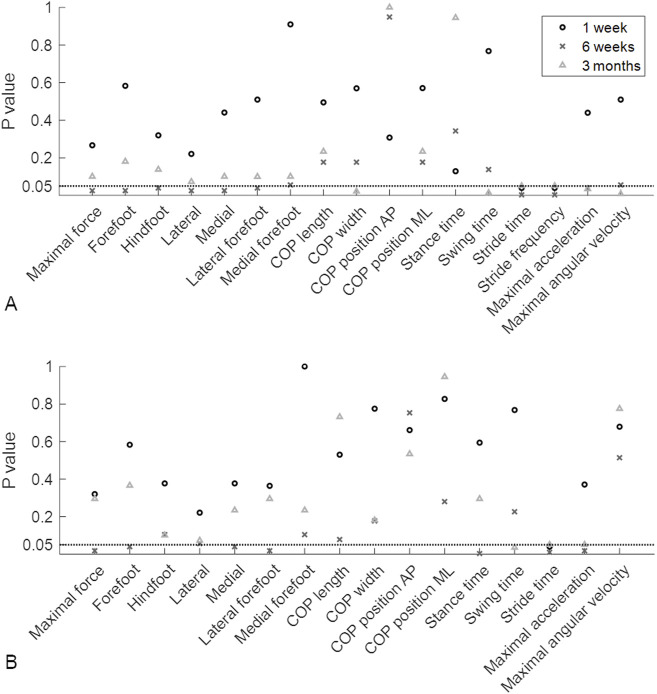
*P* values of the Mann‒Whitney U test. The *P* values indicate whether there were significant differences between patients who later developed union or nonunion at 1 week, 6 weeks and 3 months postoperatively. **(A)** *P* values of the average gait parameters. **(B)** *P* values of the asymmetry of the gait parameters. The horizontal dotted lines represent a *P* value of 0.05. AP, anteroposterior; COP, centre of pressure; ML, mediolateral.

**FIGURE 4:**
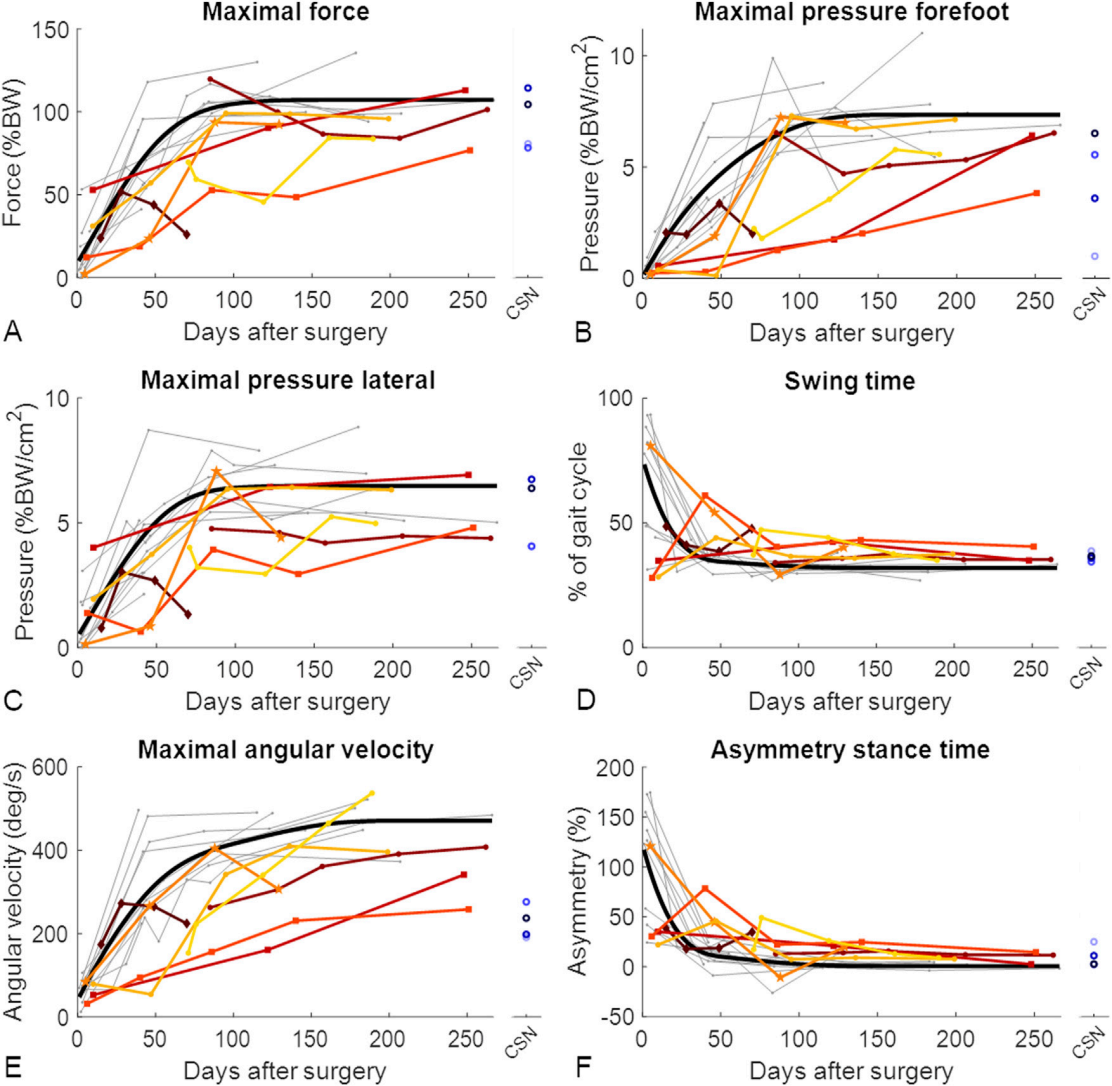
Gait improved throughout the healing phase of tibial fractures, but patients with nonunion improved slower. Each patient showing union is represented by a grey line. For visualization purposes, the optimal fit for patients who showed union is represented by the thick black line (D’Errico, 2024). Longitudinally assessed patients showing nonunion are represented by coloured lines, and cross-sectionally assessed patients showing nonunion (CSN) are represented by blue circles. The datapoints shown as squares represent the two longitudinally assessed patients with diabetes who experienced nonunion, the dark red diamond-shaped datapoints represent the patient who experienced nonunion caused by an infection, and the mid-orange pentagram-shaped data points represent the patient whose implant screws broke between the third and fourth measurements and who experienced nonunion. The gait parameters with the greatest effect sizes throughout healing are represented: **(A)** maximal force; **(B)** maximal pressure on the forefoot; **(C)** maximal pressure on the lateral side of the foot; **(D)** swinging time; **(E)** maximal angular velocity; and **(F)** asymmetry of the stance time. BW, body weight.

The ground reaction force curves showed different patterns between patients with and without union (Supplementary Figure S1). To identify these differences between the groups at different timepoints throughout the healing process, the gait parameters were compared at different timepoints using Mann‒Whitney U tests. Among all the assessed parameters at 1 week after surgery, only stance time, swing time and asymmetry of stance time showed significant differences between the LU and LN groups (*P* = 0.04 for all three parameters; p values are depicted in Figure 3, and values of the gait parameters, exact p values and effect sizes are presented in the Supplementary Material). Six weeks after surgery, 16 out of the 33 gait parameters were significantly different between the LU and LN groups. The greatest differences were detected in the pressure distribution underneath different parts of the feet and in the temporal parameters. Three months after surgery, the COP width, stride time, stride frequency, maximal angular velocity, and asymmetry of the maximal angular velocity were significantly different between the LU and LN groups (Figure 3). The differences between the groups throughout the healing process are shown in Figure 4 for the parameters with the largest effect sizes (further parameters are shown in the Supplementary Material).

Six months after surgery, there were significant differences among the LU, LN and CSN groups in terms of pressure distribution parameters, swing and stride time, maximal angular velocity, stance time asymmetry, swing time asymmetry, and maximal acceleration asymmetry (Table 2; Figure 4, Supplementary Material). *Post hoc* tests revealed that forefoot pressure, medial pressure, lateral forefoot pressure, swing time, maximal angular velocity, stance time asymmetry, swing time asymmetry, and maximal acceleration asymmetry significantly differed between patients who achieved union and both groups of patients who developed nonunion (Table 2). The temporal parameters showed group differences during all measurements throughout the healing process. The pressure distribution parameters differed during almost all measurements and seemed to be the most promising parameters for detecting patients at risk of developing nonunion.

**TABLE 2:** Results of ANOVA 6 months after surgery. Comparisons among longitudinally assessed patients who developed union (n = 7) or nonunion (n = 6) and cross-sectionally assessed patients with nonunion (n = 4) 6 months after surgery.

Gait parameter	ANOVA P value (effect size)	*Post-hoc* test LU vs. LN	*Post-hoc* test LU vs. CSN	*Post-hoc* test LN vs. CSN
Maximal force	0.069 (0.26)			
Forefoot pressure	**0.010 (0.44)**	**0.024**	**0.024**	0.989
Hindfoot pressure	0.393 (0.24)			
Lateral pressure	**0.033 (0.35)**	0.065	0.062	0.995
Medial pressure	**0.011 (0.43)**	**0.027**	**0.026**	0.991
Lateral forefoot pressure	**0.015 (0.41)**	**0.033**	**0.033**	0.988
Medial forefoot pressure	**0.024 (0.37)**	**0.047**	0.051	0.986
Centre of pressure length	0.560 (0.07)			
Centre of pressure width	0.189 (0.19)			
Centre of pressure position AP	0.241 (0.20)			
Centre of pressure position ML	0.332 (0.16)			
Stance time	0.102 (0.25)			
Swing time	**<0.001 (0.65)**	**0.012**	**<0.001**	0.295
Stride time	**0.022 (0.40)**	0.546	**0.017**	0.179
Stride frequency	0.432 (0.12)			
Maximal acceleration	0.389 (0.12)			
Maximal angular velocity	**<0.001 (0.68)**	**0.017**	**<0.001**	0.124
Asymmetry maximal force	0.447 (0.17)			
Asymmetry forefoot pressure	0.058 (0.30)			
Asymmetry hindfoot pressure	0.069 (0.28)			
Asymmetry lateral pressure	0.059 (0.30)			
Asymmetry medial pressure	0.616 (0.06)			
Asymmetry lateral forefoot pressure	0.070 (0.28)			
Asymmetry medial forefoot pressure	0.124 (0.23)			
Asymmetry centre of pressure length	0.098 (0.19)			
Asymmetry centre of pressure width	0.255 (0.16)			
Asymmetry centre of pressure position AP	0.058			
Asymmetry centre of pressure position ML	0.210			
Asymmetry stance time	**0.004 (0.51)**	**0.004**	**0.040**	0.458
Asymmetry swing time	**0.002 (0.58)**	**0.003**	**0.010**	0.837
Asymmetry stride time	0.074 (0.34)			
Asymmetry maximal acceleration	**0.010 (0.49)**	**0.002**	0.192	0.179
Asymmetry maximal angular velocity	0.408 (0.11)			

Significant ANOVA results are indicated by p values <0.05 and are presented in bold.

AP, anteroposterior; CSN, cross-sectionally assessed nonunion group; LN, longitudinally assessed nonunion group; LU, longitudinally assessed union group; ML, mediolateral.

### 3.3 Patient-reported outcome measures

**FIGURE 5:**
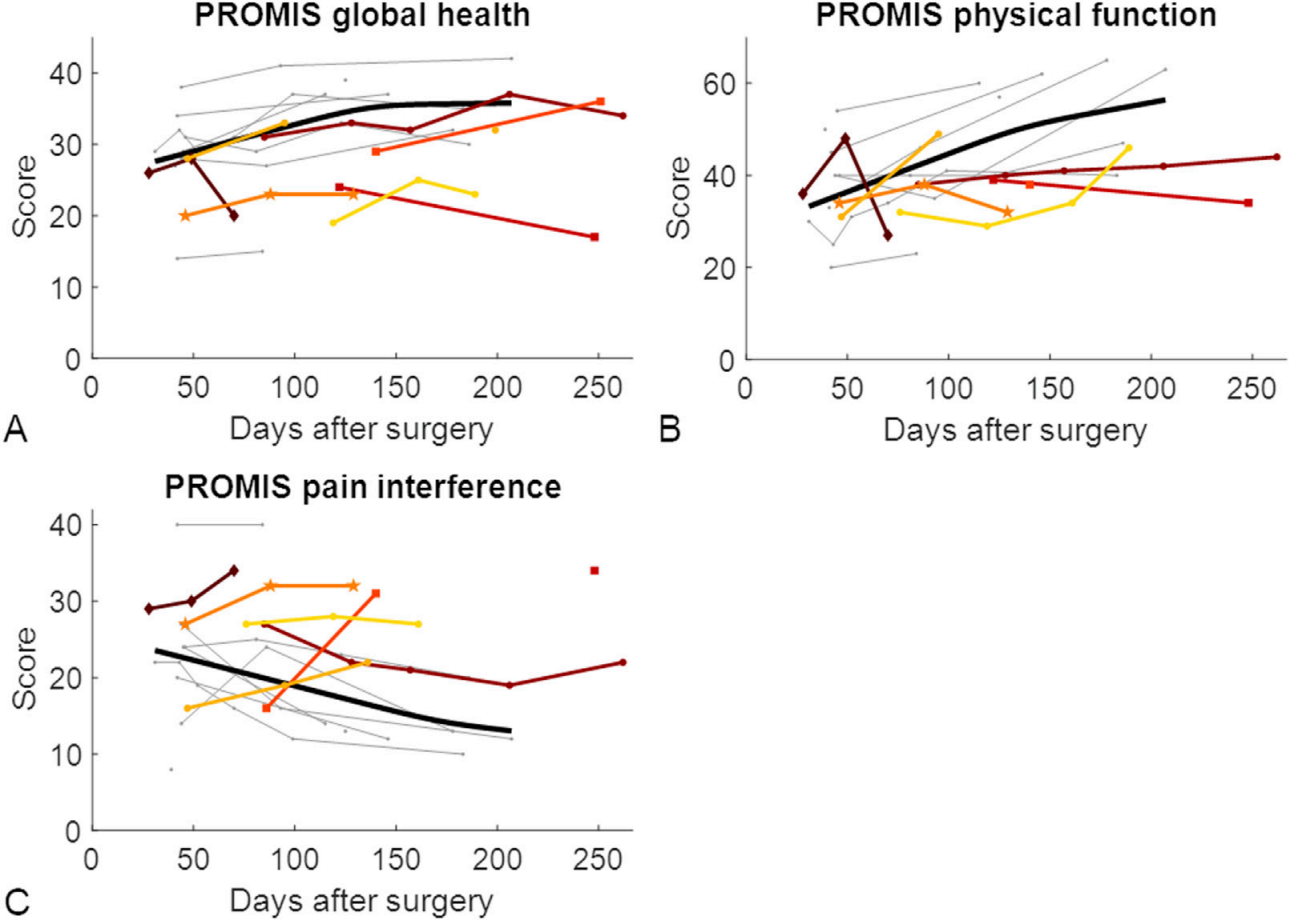
Patient-reported outcome measures throughout the healing phase of tibial shaft fractures. Each patient who developed union is represented by a grey line. For visualization purposes, the optimal fit for patients who showed union is represented by the thick black line (D’Errico, 2024). Longitudinally assessed patients showing nonunion are represented by coloured lines. The datapoints represented with squares represent the two longitudinally assessed patients with diabetes who developed nonunion, the dark red diamond-shaped datapoints represent the patient who experienced nonunion caused by an infection, and the mid-orange pentagram-shaped data points represent the patient whose implant screws broke between the third and fourth measurements and experienced nonunion. **(A)** PROMIS global health score; **(B)** PROMIS physical function score; **(C)** PROMIS pain interference score. PROMIS, Patient-reported outcomes measurement information system.

The results of the PROMIS questionnaires did not differ between the LU and LN groups at 6 weeks or 3 months after surgery. However, 6 months after surgery, the global health and physical function questionnaire results were significantly different between the LU and LN groups (Table 3; Figure 5). Since differences between the groups were only found at 6 months, there was limited benefit of using the three PROMIS questionnaires in addition to routine radiographs to detect patients at risk of developing nonunion.

**TABLE 3:** Results of the PROMIS questionnaires.

Questionnaire	P value (effect size) 6 weeks (n union = 11, n nonunion = 4)	P value (effect size) 3 months (n union = 6, n nonunion = 6)	P value (effect size) 6 months (n union = 6, n nonunion = 5)
Global health PROMIS	0.257 (−0.50)	0.314 (−0.40)	**0.044 (−0.77)**
Physical function PROMIS	0.853 (−0.11)	0.522 (−0.27)	**0.017 (−0.87)**
Pain interference PROMIS	0.516 (0.30)	0.261 (0.42)	0.051 (0.89)

Significant results are indicated by p values <0.05 and are presented in bold.

PROMIS, Patient-reported outcomes measurement information system.

## 4 Discussion

Since nonunion is a frequent complication of tibial shaft fractures and the time to diagnosis is typically 6 to 9 months, better detection methods are desirable. Therefore, the aim of this study was to determine whether it is possible to detect patients at risk of developing nonunion early in the healing process based on gait analysis via instrumented insoles or based on PROMIS. Patients who experienced nonunion had gait patterns different from those of patients whose fractures healed. These differences were most pronounced 6 weeks and 6 months after surgery and were mainly evident in the temporal (stance time, swing time and stride time) and pressure distribution parameters at the injured side. The PROMIS significantly differed between patients whose fractures healed and those who developed nonunion only at 6 months after surgery. Gait analysis can be used to detect patients at risk of developing nonunion as early as 6 weeks after surgery, which may be a great asset to clinical decision making.

Earlier detection of patients at risk of nonunion would allow earlier intervention. The inclusion of gait analyses with instrumented insoles in routine clinical practice could identify more patients at risk and may help guide surgeons in choosing the optimal intervention type and timing. When asked, trauma surgeons responded that they preferred to monitor fracture healing by using radiographs rather than performing functional assessments (Bhandari et al., 2012); however, radiography is generally delayed by a few weeks due to delayed mineralization of the callus. Among the possible reasons are limited time and evidence for the clinical benefits of functional parameters. By quantifying the outcomes of functional assessments, such as the gait analysis in this study, objective functional outcomes may aid healthcare professionals in diagnosing nonunion earlier. The benefits of these assessments, however, need to be proven in clinical studies. By administering noninvasive therapies earlier to patients who are identified as being at risk, healing times could be shortened, and nonunion could be avoided, which may improve quality of life and reduce costs for society (Hak et al., 2014; Brinker et al., 2013; Antonova et al., 2013).

The earlier the development of nonunion can be detected, the earlier additional treatment can be started. Gait analysis within the first few days after surgery is unlikely to predict healing outcomes. During this initial period, most patients put very little weight on the injured leg, possibly because of pain or fear of pain. In this study, some patients showed a severely altered gait pattern, which might have been due to difficulties with learning to walk with crutches. There were significant differences in 16 of the 33 gait parameters between the LU and LN groups at approximately 6 weeks after surgery. Based on the temporal and pressure distribution parameters, this seemed to be the first timepoint at which patients at risk of developing nonunion can be detected. At approximately 3 months, the pressure distribution parameters no longer differed between the LU and LN groups; however, the temporal and angular velocity parameters showed significant group differences. Six months after surgery, patients who experienced nonunion showed differences in both radiographic findings and gait patterns compared to patients who achieved union. This is the first study to present gait differences between patients who achieved union and multiple patients who did not achieve union. These gait differences were predominantly observed in the temporal and pressure distribution parameters.

The most promising parameters were the temporal parameters and the asymmetry of these parameters because they showed significant differences at all timepoints throughout the healing process. Another promising parameter was the maximal angular velocity during the push-off phase. At three and 6 months after surgery, this parameter significantly differed between patients who achieved union and those who did not. Earlier in the healing process, there were also marked differences in the maximal angular velocity; however, this was not the case for patients with nonunion caused by complications (infection and implant failure). Moreover, patients in the CSN group clearly had a lower maximal angular velocity than patients who achieved union. Therefore, maximal angular velocity is a promising parameter for detecting patients at risk of nonunion. A lower angular velocity at push-off results in lower push-off forces, which is commonly observed in gait disorders and ageing (Franz, 2016; Farris et al., 2015; Kempen et al., 2016) and leads to lower gait speed and energy efficiency (Pieper et al., 2021; Hsiao et al., 2016), making the maximal angular velocity an important parameter to monitor.

Certain comorbidities, such as diabetes, are known to slow fracture healing (Tanios et al., 2022). This could be observed in the gait parameters of two patients with diabetes in the LN group (indicated with squares in Figure 4 and Supplementary Material), which was reflected in lower values of the forefoot-related pressure distribution parameters. For the authors, however, decreased forefoot pressure values were unexpected, as people with diabetes are known to experience increased pressure underneath the second to fourth metatarsophalangeal joints (Falzon et al., 2017), resulting in increased pressure and the development of diabetic ulcers on the forefoot (Prompers et al., 2007). Possible explanations for this discrepancy may arise from an increased awareness of diabetic patients that they are at particular risk of developing complications once the given instructions are not followed. Another factor could be the nonrepresentation and low sample size of only two patients. Therefore, a larger cohort of diabetic patients should be investigated in future studies.

Nonunion caused by complications, such as infection or implant failure, seems to be more difficult to distinguish from union since patients might show improvements during the initial phase of healing, after which a decrease in performance might only be observed at the onset of a complication. Therefore, gait analysis might not be suitable for detecting all types of nonunion. Since two of the patients in the LN group experienced nonunion due to complications, the differences between the LU and LN groups might have been even more pronounced if these two patients were excluded from the analysis. Several gait parameters of these two patients were better than those of the other patients in the LN group at 6 weeks, when these patients were still on the regular healing trajectory before the occurrence of complications leading to nonunion.

Based on the results of the PROMIS questionnaires, the LU and LN groups were indistinguishable until 6 months after surgery. Both groups seemed to improve their PROMIS scores over time, but at 6 months, patients who experienced union had better scores than patients who experienced nonunion. Improvements in the PROMIS scores throughout the first 6 months of fracture healing were also observed in patients with a pilon fracture of the tibia (Kellam et al., 2023). Unfortunately, not all patients were willing or able to complete the questionnaires, or they left questions unanswered, leading to several missing data points. The PROMIS questionnaires might be useful for analysing how patients perceive their health status but are not specific enough to distinguish between union and nonunion. Additionally, missing data need to be taken into account.

One of the limitations of this study is that only 22 patients were assessed. However, this included seven longitudinally assessed nonunion patients and four cross-sectionally assessed nonunion patients, which is a rather large number of patients experiencing nonunion considering that approximately 7.6% of tibial shaft fractures result in nonunion (Zura et al., 2016). This is likely caused by the more severe cases and patients with several comorbidities that are treated in the University Hospital. Due to differences in the timing of outpatient visits, the sizes of the groups differed in the statistical analyses, making it difficult to compare the results between visits. These limitations could be overcome in a larger multicentre study.

### 4.1 Clinical application

Gait analysis with instrumented insoles is a promising tool to detect patients at risk of nonunion early on, while according to the findings of this study, PROMIS cannot be used for this purpose. The most suitable gait parameters to predict nonunion according to the present study are medial and lateral pressure, (asymmetry of) temporal parameters, rotation velocity of the foot around the mediolateral axis at initial contact and the asymmetry of the maximal acceleration of the feet. The instrumented insoles are an easy-to-use and relatively inexpensive measurement tool and can therefore be easily implemented in routine clinical care. In addition, instrumented insoles can serve to monitor patients continuously throughout their daily life (Warmerdam et al., 2024a). This approach seems to allow earlier detection of healing complications than laboratory-based gait measurements. When analysing insole data, the age, body weight, body mass index, body height and hand grip strength of the patient (Wolff et al., 2023), as well as the walking surface (Warmerdam et al., 2024b) and slope (Wolff et al., 2024) influence the data in characteristic ways. During the first week after a lower limb fracture when the ground-reaction force curve is still heavily altered, the highest overall force, the mean force and absolute time between inflection points of the force curve seem to be useful parameters to analyse the insole data (Wolff et al., 2025). On the other hand, a standing test with instrumented insoles alone that assesses changes in the pressure distribution under the feet was shown not to be suitable for the prediction of union vs. nonunion after tibial fractures (Warmerdam et al., 2024c).

## 5 Conclusion

This study is the first to show that gait analysis with instrumented insoles can be used to detect patients at high risk of developing nonunion of a tibial shaft fracture. Gait analysis detected these patients earlier than was possible based on radiographs and PROMIS. Hence, additional therapy to stimulate fracture healing can be initiated earlier, thereby decreasing the incidence of nonunion and all its negative consequences. These very promising results should be confirmed in a larger multicentre study before gait analysis can be implemented in routine clinical care for the monitoring of tibial shaft fractures after surgery.

## Data Availability

The data set analysed during the present study is available from the corresponding author on reasonable request. Access may be granted based on a collaboration agreement. The requesting institution needs to fall within the eligibility criteria of German data protection law.
