# Supplementary material for: Gait analysis with smart insoles can identify patients at risk of tibial shaft fracture nonunion as early as six weeks after surgery: longitudinal and cross-sectional study
Source: Front Bioeng Biotechnol. 2025 Jun 25;13:1536738. doi: 10.3389/fbioe.2025.1536738 (PMC12238006; doi:10.3389/fbioe.2025.1536738)
Supplement: Supplementary file 1 [file DataSheet1.pdf]

**Supplementary material**

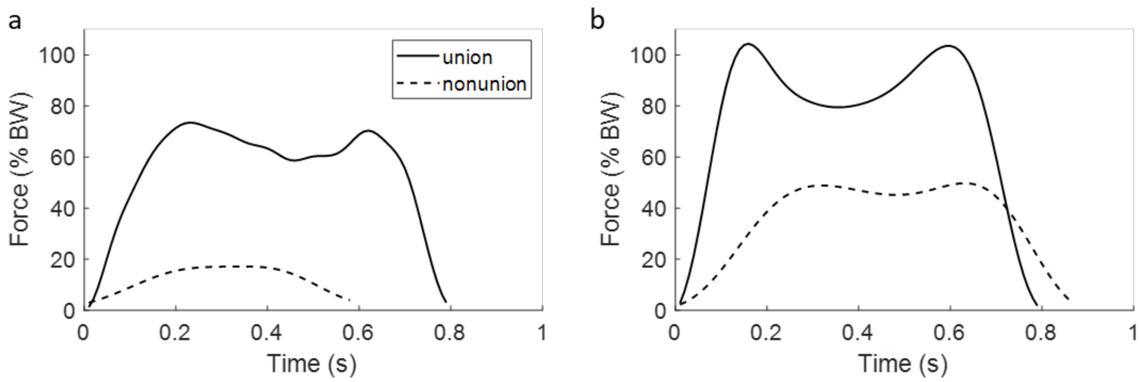


Figure 1. Vertical ground reaction force curves during the stance phase of a patient who showed union and a patient who showed nonunion six weeks (a) and three months (b) after surgery.

Table 1. Gait parameters of patients with union and nonunion at six weeks and three months after surgery. P values < 0.05 are presented in bold.

| **Gait parameter** | **Union group at 1 week** | **Nonunion group at 1 week** | **Union group at 6 weeks** | **Nonunion group at 6 weeks** | **Union group at 3 months** | **Nonunion group at 3 months** |
|---|---|---|---|---|---|---|
| Maximal force (%BW) | 13.9 ± 16.5 | 24.5 ± 19.3 | 73.2 ± 21.6 | **35.8** ± 17.9 | 105.0 ± 14.1 | 72.8 ± 36.5 |
| Forefoot pressure (%BW/cm$^2$) | 0.5 ± 0.6 | 0.7 ± 0.8 | 4.3 ± 2.0 | **1.4** ± 1.5 | 7.3 ± 1.6 | 4.7 ± 2.7 |
| Hindfoot pressure (%BW/cm$^2$) | 1.3 ± 2.1 | 2.3 ± 2.3 | 7.5 ± 2.0 | **3.7** ± 2.9 | 10.3 ± 1.7 | 7.2 ± 3.6 |
| Lateral pressure (%BW/cm$^2$) | 0.9 ± 1.6 | 1.7 ± 1.5 | 4.4 ± 1.8 | **2.0** ± 1.5 | 6.6 ± 0.8 | 4.4 ± 2.1 |
| Medial pressure (%BW/cm$^2$) | 0.6 ± 0.8 | 0.9 ± 0.7 | 3.5 ± 1.3 | **2.0** ± 0.7 | 5.3 ± 1.4 | 3.5 ± 1.6 |
| Lateral forefoot pressure (%BW/cm$^2$) | 0.7 ± 1.0 | 0.7 ± 0.5 | 5.2 ± 2.6 | **1.5** ± 1.8 | 8.8 ± 2.5 | 5.7 ± 3.2 |
| Medial forefoot pressure (%BW/cm$^2$) | 0.5 ± 0.6 | 0.7 ± 1.3 | 5.4 ± 2.8 | 1.9 ± 2.1 | 8.7 ± 2.7 | 5.4 ± 3.4 |
| Centre of pressure length (AU) | 0.21 ± 0.24 | 0.30 ± 0.21 | 0.65 ± 0.06 | 0.44 ± 0.22 | 0.62 ± 0.05 | 0.63 ± 0.16 |

| | | | | | | |
|---|---|---|---|---|---|---|
| Centre of pressure width (AU) | 0.11 ± 0.12 | 0.16 ± 0.11 | 0.17 ± 0.07 | 0.13 ± 0.05 | **0.16** ± 0.04 | **0.34** ± 0.06 |
| Centre of pressure position AP (AU) | 0.51 ± 0.11 | 0.44 ± 0.17 | 0.44 ± 0.07 | 0.45 ± 0.15 | 0.45 ± 0.05 | 0.45 ± 0.10 |
| Centre of pressure position ML (AU) | 0.45 ± 0.06 | 0.44 ± 0.09 | 0.43 ± 0.03 | 0.46 ± 0.04 | 0.41 ± 0.03 | 0.43 ± 0.02 |
| Stance time (%) | **30.9** ± 23.1 | **55.8** ± 22.2 | **63.7** ± 3.1 | **50.6** ± 10.1 | 68.8 ± 2.4 | 61.3 ± 6.8 |
| Swing time (%) | **69.1** ± 23.1 | **44.2** ± 22.2 | **36.3** ± 3.1 | **49.4** ± 10.1 | 31.2 ± 2.4 | 38.7 ± 6.8 |
| Stride time (s) | 3.31 ± 1.49 | 2.58 ± 1.06 | 1.40 ± 0.29 | **2.11** ± 0.92 | **1.25** ± 0.12 | **1.43** ± 0.14 |
| Stride frequency (strides/min) | 25.7 ± 10.4 | 31.9 ± 13.7 | 55.1 ± 11.5 | 39.1 ± 14.3 | **59.8** ± 5.4 | **51.0** ± 5.1 |
| Maximal acceleration (g) | 1.17 ± 0.17 | 1.34 ± 0.25 | 2.47 ± 0.99 | 1.87 ± 0.35 | 2.45 ± 0.45 | 2.52 ± 1.02 |
| Maximal angular velocity (°/s) | 68.1 ± 30.4 | 84.4 ± 54.3 | 330.3 ± 101.0 | 170.1 ± 111.8 | **408.9** ± 45.0 | **288.4** ± 91.1 |
| Asymmetry maximal force (%) | 149.6 ± 46.4 | 106.0 | 30.9 ± 31.7 | **97.5** ± 47.2 | -11.1 ± 36.7 | 48.2 ± 57.9 |
| Asymmetry forefoot pressure (%) | 163.7 ± 31.4 | 147.0 ± 41.2 | 49.8 ± 47.1 | **139.3** ± 58.7 | -0.2 ± 45.9 | 56.1 ± 75.6 |
| Asymmetry hindfoot pressure (%) | 152.1 ± 64.2 | 110.4 ± 89.4 | 27.8 ± 34.5 | 95.6 ± 67.6 | -13.9 ± 32.1 | 42.9 ± 73.8 |
| Asymmetry lateral pressure (%) | 151.0 ± 48.8 | 106.1 ± 81.2 | 28.9 ± 39.5 | 105.2 ± 67.4 | -13.9 ± 32.1 | 41.4 ± 63.5 |
| Asymmetry medial pressure (%) | 153.7 ± 43.1 | 121.8 ± 62.8 | 41.4 ± 41.7 | **95.3** ± 26.7 | 1.2 ± 45.2 | 54.5 ± 58.2 |
| Asymmetry lateral forefoot pressure (%) | 164.2 ± 38.0 | 141.0 ± 47.8 | 39.3 ± 50.6 | **139.2** ± 61.9 | -7.1 ± 47.4 | 47.9 ± 77.1 |

| | | | | | | |
|---|---|---|---|---|---|---|
| Asymmetry medial forefoot pressure (%) | 169.2 ± 36.9 | 173.5 ± 38.6 | 52.5 ± 52.5 | 137.7 ± 63.7 | 7.9 ± 50.7 | 64.6 ± 79.3 |
| Asymmetry centre of pressure length (%) | 118.7 ± 73.0 | 80.0 ± 69.7 | 3.5 ± 8.5 | 47.7 ± 44.5 | 4.9 ± 7.0 | 12.1 ± 29.7 |
| Asymmetry centre of pressure width (%) | 90.1 ± 95.9 | 61.0 ± 82.9 | 11.7 ± 53.2 | 56.2 ± 51.3 | 19.8 ± 28.1 | -2.1 ± 23.5 |
| Asymmetry centre of pressure position AP (%) | -1.4 ± 26.0 | 8.4 ± 28.1 | 11.5 ± 20.7 | 19.8 ± 31.7 | 8.2 ± 16.7 | 16.2 ± 19.2 |
| Asymmetry centre of pressure position ML (%) | -4.7 ± 13.5 | -3.3 ± 15.4 | 2.2 ± 7.9 | -4.1 ± 11.6 | 2.6 ± 7.1 | 3.8 ± 6.4 |
| Asymmetry stance time (%) | **104.9** ± 58.8 | **49.4** ± 40.4 | **12.5** ± 10.7 | **46.9** ± 24.4 | -1.3 ± 11.5 | 15.4 ± 16.0 |
| Asymmetry swing time (%) | -137.4 ± 43.7 | -115.0 ± 39.9 | -29.7 ± 30.2 | **-91.2** ± 47.3 | 1.4 ± 21.9 | -29.1 ± 29.4 |
| Asymmetry stride time (%) | 0.1 ± 0.4 | 0.2 ± 0.3 | -0.0 ± 0.1 | -0.0 ± 0.1 | 0.0 ± 0.0 | 0.0 ± 0.0 |
| Asymmetry maximal acceleration (%) | 33.4 ± 15.8 | 49.7 ± 41.5 | 6.5 ± 9.9 | **41.7** ±17.1 | 2.5 ± 10.7 | 13.4 ± 30.9 |
| Asymmetry maximal angular velocity (%) | 94.8 ± 36.8 | 104.1 ± 39.5 | 13.9 ± 18.5 | 51.6 ± 43.4 | **-0.6** ± 11.3 | **20.7** ± 16.7 |

*AP = anteroposterior; AU = arbitrary units; BW = body weight; ML = mediolateral*

Table 2. Gait parameters of the three patient groups at six months after surgery. P values < 0.05 are presented in bold.

| **Gait parameter** | **Union** | **Longitudinal nonunion** | **Cross-sectional nonunion** |
|---|---|---|---|
| Maximal force (%BW) | 102.7 ± 14.8 | 82.4 ± 17.3 | 94.4 ± 17.7 |
| Forefoot pressure (%BW/cm$^2$) | 6.9 ± 2.2 | **4.8** ± 2.4 | 4.2 ± 2.4 |
| Hindfoot pressure (%BW/cm$^2$) | 10.1 ± 2.0 | **8.2** ± 2.1 | 8.0 ±1.6 |
| Lateral pressure (%BW/cm$^2$) | 6.5 ± 1.3 | **4.9** ± 1.3 | 5.3 ± 1.4 |
| Medial pressure (%BW/cm$^2$) | 4.9 ± 1.7 | **3.8** ± 1.2 | 3.5 ± 0.9 |
| Lateral forefoot pressure (%BW/cm$^2$) | 9.3 ± 3.2 | **5.3** ± 2.8 | 5.4 ± 2.3 |

| | | | |
|---|---|---|---|
| Medial forefoot pressure (%BW/cm$^2$) | 8.1 ± 3.9 | 5.8 ± 3.1 | 4.7 ± 3.0 |
| Centre of pressure length (AU) | 0.66 ± 0.02 | 0.68 ± 0.07 | 0.62 ± 0.16 |
| Centre of pressure width (AU) | 0.20 ± 0.06 | 0.19 ± 0.04 | **0.23** ± 0.08 |
| Centre of pressure position AP (AU) | 0.46 ± 0.04 | 0.42 ± 0.07 | 0.38 ± 0.11 |
| Centre of pressure position ML (AU) | 0.40 ± 0.03 | 0.43 ± 0.04 | 0.41 ± 0.03 |
| Stance time (%) | **67.3** ± 3.9 | **61.1** ± 3.5 | 63.5 ± 1.8 |
| Swing time (%) | **32.7** ± 3.9 | **38.9** ± 3.5 | 36.5 ± 1.8 |
| Stride time (s) | 1.16 ± 0.08 | **1.29** ± 0.10 | **1.44** ± 0.16 |
| Stride frequency (strides/min) | 64.8 ± 4.8 | 56.7 ± 4.4 | **61.0** ± 16.6 |
| Maximal acceleration (g) | 3.39 ± 1.14 | 2.76 ± 1.10 | 2.85 ± 0.88 |
| Maximal angular velocity (°/s) | 463.9 ± 48.6 | 337.1 ± 133.9 | **225.7** ±39.4 |
| Asymmetry maximal force (%) | 3.2 ± 12.0 | **21.1** ± 27.7 | 16.8 ± 14.3 |
| Asymmetry forefoot pressure (%) | 16.3 ± 28.5 | **51.2** ± 50.6 | 62.4 ± 60.0 |
| Asymmetry hindfoot pressure (%) | 5.1 ± 17.8 | 22.7 ± 18.9 | 10.8 ± 8.7 |
| Asymmetry lateral pressure (%) | -0.1 ± 10.1 | 23.7 ± 34.2 | 14.3 ± 15.8 |
| Asymmetry medial pressure (%) | 19.6 ± 30.2 | **31.3** ± 27.2 | 34.1 ± 24.7 |
| Asymmetry lateral forefoot pressure (%) | 1.2 ± 24.7 | **5**6.0 ± 52.3 | 61.1 ± 52.2 |
| Asymmetry medial forefoot pressure (%) | 31.1 ± 46.8 | 52.4 ± 48.8 | 68.7 ± 68.9 |
| Asymmetry centre of pressure length (%) | 2.4 ± 2.9 | 3.2 ± 13.0 | 19.8 ± 30.6 |
| Asymmetry centre of pressure width (%) | -5.0 ± 32.5 | 28.5 ± 21.7 | -8.6 ± 39.0 |
| Asymmetry centre of pressure position AP (%) | 8.5 ± 6.9 | 23.1 ± 16.1 | 36.6 ± 28.7 |
| Asymmetry centre of pressure position ML (%) | 8.0 ± 7.8 | -0.9 ± 11.5 | 0.1 ± 6.3 |
| Asymmetry stance time (%) | **3.0** ± 5.5 | **15.7** ± 7.0 | 10.3 ± 10.6 |
| Asymmetry swing time (%) | -6.1 ± 11.2 | **-30.4** ± 12.3 | -22.8 ± 25.3 |
| Asymmetry stride time (%) | 0.01 ± 0.04 | 0.01 ± 0.03 | -0.08 ± 0.11 |
| Asymmetry maximal acceleration (%) | -2.1 ± 10.9 | **29.5** ± 11.5 | 13.4 ± 25.9 |
| Asymmetry maximal angular velocity (%) | 4.3 ± 12.5 | 9.2 ± 13.3 | **-19.0** ± 37.1 |

*AP = anteroposterior; AU = arbitrary units; BW = body weight; ML = mediolateral*

Table 3. Comparison between longitudinally assessed patients showing union and those showing nonunion at one week and three months after surgery. P values and effect sizes of Mann–Whitney U tests; p values < 0.05 are presented in bold.

| **Gait parameter** | **P value (effect size) 1 week** (n union 11, n nonunion 5) | **P value (effect size) 6 weeks** (n union 11, n nonunion 4) | **P value (effect size) 3 months** (n union 7, n nonunion 5) |
|---|---|---|---|
| Maximal force | 0.267 (0.38) | **0.026 (-0.77)** | 0.101 (-0.57) |
| Forefoot pressure | 0.583 (0.20) | **0.026 (-0.77)** | 0.181 (-0.48) |
| Hindfoot pressure | 0.320 (0.35) | **0.040 (-0.73)** | 0.138 (-0.52) |
| Lateral pressure | 0.221 (0.42) | **0.026 (-0.77)** | 0.073 (-0.62) |
| Medial pressure | 0.441 (0.27) | **0.026 (-0.77)** | 0.101 (-0.57) |
| Lateral forefoot pressure | 0.510 (0.24) | **0.040 (-0.73)** | 0.100 (-0.57) |
| Medial forefoot pressure | 0.910 (-0.06) | 0.056 (-0.68) | 0.101 (-0.57) |
| Centre of pressure length | 0.495 (0.24) | 0.177 (-0.50) | 0.234 (0.43) |
| Centre of pressure width | 0.570 (0.20) | 0.177 (-0.50) | **0.022 (0.76)** |
| Centre of pressure position AP | 0.308 (-0.35) | 0.949 (0.05) | 1.000 (0.00) |
| Centre of pressure position ML | 0.571 (-0.20) | 0.177 (0.50) | 0.234 (0.43) |
| Stance time | **0.040 (0.68)** | **0.003 (-0.96)** | 0.051 (-0.67) |
| Swing time | **0.040 (-0.68)** | **0.003 (0.96)** | 0.051 (0.67) |
| Stride time | 0.440 (-0.28) | **0.040 (0.73)** | **0.035 (0.71)** |
| Stride frequency | 0.510 (0.24) | 0.056 (-0.68) | **0.008 (-0.86)** |
| Maximal acceleration | 0.129 (0.52) | 0.343 (-0.36) | 0.945 (-0.05) |
| Maximal angular velocity | 0.768 (0.12) | 0.138 (-0.55) | **0.014 (-0.81)** |
| Asymmetry maximal force | 0.320 (-0.35) | **0.018 (0.82)** | 0.295 (0.38) |
| Asymmetry forefoot pressure | 0.583 (-0.20) | **0.040 (0.73)** | 0.366 (0.33) |
| Asymmetry hindfoot pressure | 0.377 (-0.31) | 0.104 (0.59) | 0.101 (-0.57) |
| Asymmetry lateral pressure | 0.221 (-0.42) | 0.056 (0.68) | 0.073 (0.62) |
| Asymmetry medial pressure | 0.377 (-0.31) | **0.040 (0.73)** | 0.234 (0.43) |
| Asymmetry lateral forefoot pressure | 0.364(-0.31) | **0.018 (0.82)** | 0.295 (0.38) |
| Asymmetry medial forefoot pressure | 1.000 (0.02) | 0.104 (0.60) | 0.234 (0.43) |
| Asymmetry centre of pressure length | 0.530 (-0.22) | 0.078 (0.64) | 0.731 (-0.14) |
| Asymmetry centre of pressure width | 0.775 (-0.11) | 0.177 (0.50) | 0.181 (0.48) |
| Asymmetry centre of pressure position AP | 0.661 (0.16) | 0.753 (0.14) | 0.534 (0.24) |

| | | | |
|---|---|---|---|
| Asymmetry centre of pressure position ML | 0.827 (0.09) | 0.280 (-0.41) | 0.945 (0.05) |
| Asymmetry stance time | **0.040 (-0.68)** | **0.010 (0.86)** | 0.051 (0.67) |
| Asymmetry swing time | 0.371 (0.32) | **0.018 (-0.82)** | 0.051 (-0.67) |
| Asymmetry stride time | 0.679 (0.16) | 0.601 (-0.25) | 0.775 (-0.12) |
| Asymmetry maximal acceleration | 0.594 (0.20) | **0.003 (0.96)** | 0.295 (0.39) |
| Asymmetry maximal angular velocity | 0.768 (0.12) | 0.226 (0.46) | **0.035 (0.71)** |

*AP = anteroposterior; ML = mediolateral*

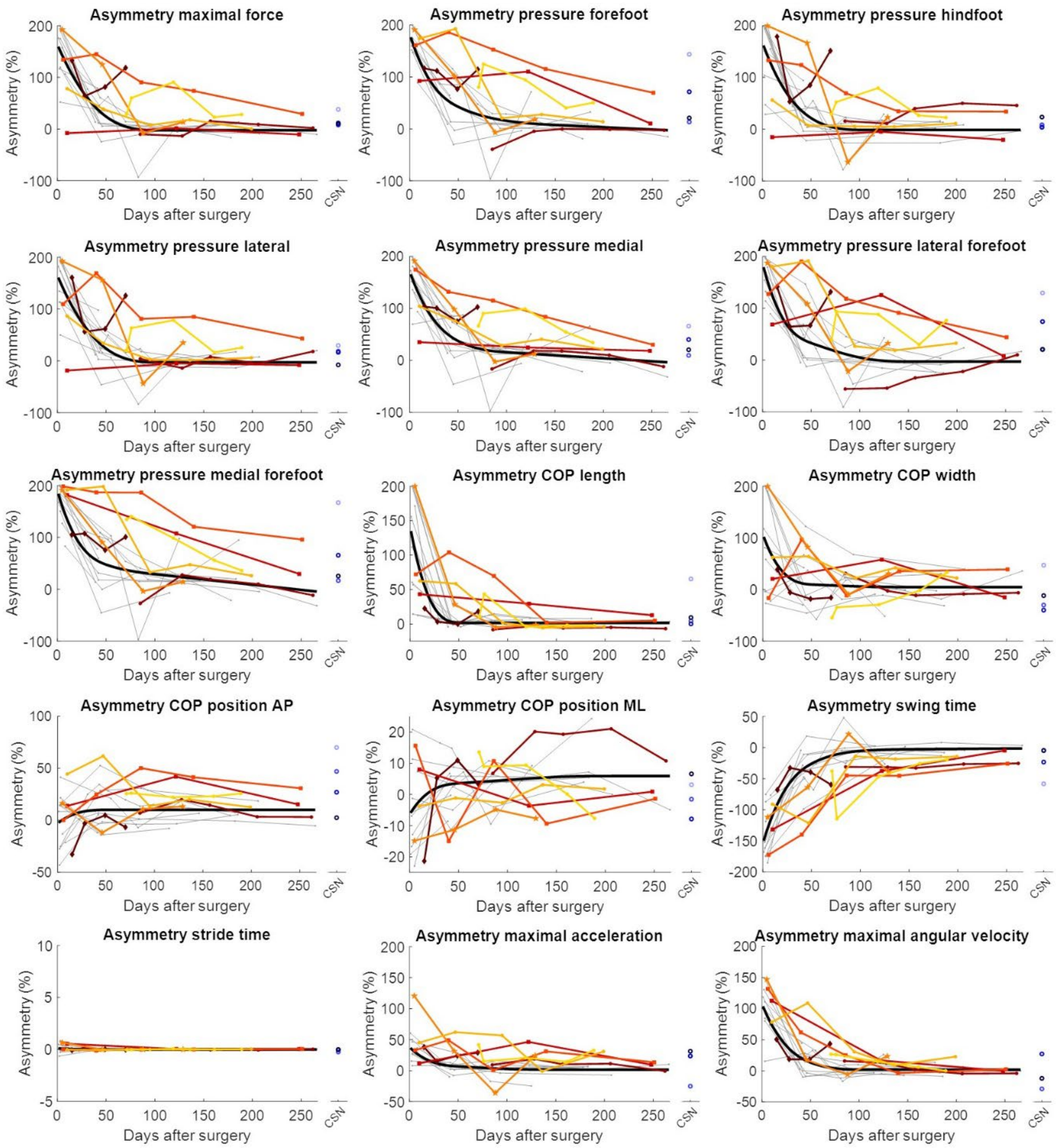


Figure 2. Gait parameters throughout the healing phase of tibial fractures that are less suitable for detecting patients at risk of developing nonunion. Each patient showing union is represented by a grey line. For visualization purposes, the optimal fit for patients who showed union is represented by the thick black line. Longitudinally assessed patients showing nonunion are represented by coloured lines, and cross-sectionally assessed patients showing nonunion (CSN) are represented by blue circles. The datapoints represented with squares represent the two longitudinally assessed patients with diabetes who exhibited nonunion, the dark red diamond-shaped datapoints represent the patient who exhibited nonunion caused by an infection, and the mid-

orange pentagram-shaped data points represent the patient whose implant screws broke between the third and fourth measurements and exhibited nonunion. The gait parameters with the greatest effect sizes throughout healing are represented: a. maximal force; b. maximal pressure on the forefoot; c. maximal pressure on the lateral side of the foot; d. swinging time; e. maximal angular velocity; and f. asymmetry of the stance time. BW = body weight.
